# Effects of krill oil and lean and fatty fish on cardiovascular risk markers: a randomised controlled trial

**DOI:** 10.1017/jns.2017.64

**Published:** 2018-01-17

**Authors:** Amanda Rundblad, Kirsten B. Holven, Inge Bruheim, Mari C. Myhrstad, Stine M. Ulven

**Affiliations:** 1 Department of Nursing and Health Promotion, Faculty of Health Sciences, Oslo and Akershus University College of Applied Sciences, PO Box 4 St. Olavs plass, 0130 Oslo, Norway; 2 Department of Nutrition, Institute for Basic Medical Sciences, University of Oslo, PO Box 1046 Blindern, 0317 Oslo, Norway; 3 National Advisory Unit on Familial Hypercholesterolemia, Department of Endocrinology, Morbid Obesity and Preventive Medicine, Oslo University Hospital, PO Box 4950 Nydalen, 0424 Oslo, Norway; 4 Rimfrost AS, N-6099 Fosnavaag, Norway

**Keywords:** Fish, Krill oil, EPA, Docosapentaenoic acid, DHA, TAG, Lipoprotein subclasses, DPA, docosapentaenoic acid, HOSO, high-oleic sunflower oil, IQR, interquartile range, XS-VLDL, smallest VLDL subclass

## Abstract

Fish consumption and supplementation with *n*-3 fatty acids reduce CVD risk. Krill oil is an alternative source of marine *n*-3 fatty acids and few studies have investigated its health effects. Thus, we compared krill oil supplementation with the intake of fish with similar amounts of *n*-3 fatty acids on different cardiovascular risk markers. In an 8-week randomised parallel study, thirty-six healthy subjects aged 18–70 years with fasting serum TAG between 1·3 and 4·0 mmol/l were randomised to receive either fish, krill oil or control oil. In the fish group, subjects consumed lean and fatty fish, according to dietary guidelines. The krill and control group received eight capsules per d containing 4 g oil per d. The weekly intake of marine *n*-3 fatty acids from fish given in the fish group and from krill oil in the krill group were 4103 and 4654 mg, respectively. Fasting serum TAG did not change between the groups. The level of total lipids (*P* = 0·007), phospholipids (*P* = 0·015), cholesterol (*P* = 0·009), cholesteryl esters (*P* = 0·022) and non-esterified cholesterol (*P* = 0·002) in the smallest VLDL subclass increased significantly in response to krill oil supplementation. Blood glucose decreased significantly (*P* = 0·024) in the krill group and vitamin D increased significantly in the fish group (*P* = 0·024). Furthermore, plasma levels of marine *n*-3 fatty acids increased significantly in the fish and krill groups compared with the control (all *P* ≤ 0·0003). In conclusion, supplementation with krill oil and intake of fish result in health-beneficial effects. Although only krill oil reduced fasting glucose, fish provide health-beneficial nutrients, including vitamin D.

Intake of the marine *n*-3 fatty acids EPA (20 : 5*n*-3) and DHA (22 : 6*n*-3) from fish and fish oil has been reported to reduce the risk of CVD and CVD death^(^^1^^–^^7^^)^. However, contradictory results exist regarding the beneficial effects of marine *n*-3 fatty acids^(^^8^^,^^9^^)^. In a recent meta-analysis of randomised controlled trials, no overall significant reduction in CHD risk with EPA and DHA supplementation was observed; nonetheless, there was a significant risk reduction for subjects with elevated TAG^(^^10^^)^. Indeed, the CVD risk reduction effect is thought to be mainly mediated by reduction of serum TAG, in addition to a range of other mechanisms such as lowering of blood pressure, plaque-stabilising and anti-arrhythmic and anti-inflammatory effects^(^^11^^–^^16^^)^. The TAG-lowering effect of marine *n*-3 fatty acids is dependent on the dose as well as baseline TAG levels^(^^17^^)^. Hence, persons with elevated TAG are thought to have the greatest benefit of consuming marine *n*-3 fatty acids, which have been used in the treatment of hypertriacylglycerolaemia^(^^18^^)^.

In addition to containing EPA and DHA, fish is a good source of iodine, Se, taurine, high-quality proteins, and vitamins D and B_12_^(^^19^^)^. Hence, dietary guidelines recommend increasing the consumption of fish, with an emphasis on consuming fatty fish^(^^20^^–^^22^^)^. However, intake of lean fish is also recommended as it may reduce blood pressure^(^^23^^)^ and lower TAG levels^(^^24^^–^^26^^)^. These effects are most probably not only mediated by EPA and DHA, as lean fish contains low amounts of *n*-3 fatty acids. Rather, lean fish protein and bioactive peptides have been suggested to mediate the beneficial effects observed^(^^23^^,^^25^^)^.

As fish and fish oil are limited resources, there has been growing interest for exploiting alternative sources of marine *n*-3 fatty acids. Krill oil is a good source of marine *n*-3 fatty acids. In addition, Antarctic krill (*Euphausia superba*) is the largest biomass of zooplankton, with an annual catch of about 2 % of the catch limit^(^^27^^)^. In fish and fish oil, EPA and DHA occur almost exclusively in TAG form, while in krill oil up to 65 % of EPA and DHA occur in phospholipids^(^^28^^)^. Krill oil has been shown to reduce serum TAG in some studies, in a dose-dependent manner^(^^29^^–^^32^^)^. Nevertheless, clear evidence of a TAG-lowering effect is still lacking. Although the interest for krill oil as an alternative *n*-3 supplement is rising, relatively few studies have investigated the health effects of krill oil supplementation in humans^(^^33^^)^.

Lipoproteins, including the TAG-rich chylomicrons and VLDL and the cholesterol-rich intermediate-density lipoprotein, LDL and HDL, can be divided into subclasses based on their size and density. Some subclasses are to a greater degree associated with CVD, with small dense LDL having the strongest relationship with increased CVD risk^(^^34^^)^. In addition, large VLDL and small HDL subclasses have been associated with the severity of coronary artery disease^(^^35^^)^. With the use of NMR spectroscopy, lipoprotein subclasses and their lipid constituents can be analysed. This enables a more detailed study of lipoprotein metabolism and the detection of subtle changes in the distribution of lipoproteins that is not available from standard lipid panels^(^^36^^)^. Fish consumption is shown to increase HDL particle size and the concentration of lipids in the largest HDL subclasses^(^^37^^,^^38^^)^, while fish oil supplementation has been shown to reduce VLDL size and the concentration of the largest VLDL subclass^(^^39^^)^. However, it is not known how intake of krill oil affects lipoprotein subclasses and their lipid constituents.

Our objective was to compare the health effects of intake of lean and fatty fish according to dietary guidelines, with the intake of krill oil with similar *n*-3 fatty acid content, and a control oil. We investigated the effects on the lipid profile including lipoprotein subclasses and on other CVD risk markers in individuals with slightly elevated serum TAG.

## Methods

### Subjects

Healthy men and women aged 18–70 years living in the Skedsmo municipality, Akershus county, Norway were recruited by invitation by post using addresses extracted from the National Registry with permission from the Directorate of Taxes. In addition, we recruited subjects by targeted advertisements in social media as well as advertising in local newspapers and at local companies. We interviewed those who responded by telephone, and subjects with a habitual consumption of fatty fish less than one dinner serving per week were invited to a screening visit. Participants with a stable weight (±5 %) the past 3 months, C-reactive protein < 10 mg/l, BMI between 18·5 and 35 kg/m^2^ and fasting serum TAG between 1·3 and 4·0 mmol/l were included. Initially, we wanted to recruit subjects with fasting serum TAG between 1·7 and 4·0 mmol/l, but we changed the lower limit to 1·3 mmol/l in order to include more participants. Exclusion criteria were any chronic disease, including diabetes types 1 and 2 and CVD or cancer the past 6 months, pregnancy or lactation, levels of thyroid-stimulating hormone or free thyroxine (T4) and free triiodothyronine (T3) outside the reference ranges, total cholesterol >7·8 mmol/l, hypertension (≥160/100 mmHg), planned weight reduction, excessive alcohol consumption (>40 g/d) and intake of plant sterols. Use of medications that might affect TAG levels was not allowed, except statins provided a stable dose the past 3 months. Hormonal treatment was an exclusion criterion; however, thyroxine replacement therapy and contraceptives were allowed if the administered dose was stable the past 3 months prior to inclusion and during the study.

This study was conducted according to the guidelines laid down in the Declaration of Helsinki and all procedures involving human subjects were approved by the Regional Ethics Committee for Medical Research in South East Norway (2015/706/REK sør-øst C). Written informed consent was obtained from all subjects. The study was registered at http://www.clinicaltrials.gov (ClinicalTrials.gov identifier: NCT02568228).

### Study design

An 8-week randomised controlled parallel study with three intervention groups was conducted at Oslo and Akershus University College between October 2015 and November 2016. At baseline, participants were allocated to one of the three intervention groups. The fish group (*n* 12) received three weekly fish meals; one lean fish dinner containing 140 g cod filet, one fatty fish dinner containing 140 g salmon filet and one fatty fish bread spread containing 66 g mackerel. The dinner meals were commercially available vacuum-packaged ready-made dishes for the participants to heat and eat at home. The krill group (*n* 12) and the control group (*n* 12) received eight capsules per d containing 4 g per d of krill oil (RIMFROST Sublime^®^, batch 11335; Rimfrost AS) or high-oleic sunflower oil (HOSO). Participants were instructed to take four capsules with their breakfast and four capsules with their dinner meals.

Participants were asked to refrain from dietary supplements, unless prescribed by their general practitioner. Consumption of *n*-3 supplements was not allowed during the study, and participants who were regularly taking *n*-3 supplements were asked to stop supplementation 4 weeks prior to the baseline visit. Consumption of fish was restricted to one meal of lean fish per week from the screening visit and throughout the intervention. The restriction on lean fish also applied to the fish group from the screening to the baseline visit. Subjects were instructed to not change their lifestyle habits and to maintain their habitual diet and level of physical activity during the intervention. Information about the subjects’ background diet was collected at the screening visit using a validated FFQ^(^^40^^)^.

Blood samples were drawn at baseline and at the end of study. At the same visits, the participants performed a 30-s chair stand test and a hand grip strength test. Between these visits, participants met every 14th day to collect more capsules or fish meals. On these visits, adverse events and the weight of the participants were registered.

### Description of capsules and fish meals

The fatty acid content of the krill oil and control oil capsules was analysed, as well as one randomly selected fish filet from each of the three fish meals once every 3 months, at a routine food analysis laboratory (Eurofins). The average weekly intake of marine *n*-3 fatty acids was 4103 mg from lean and fatty fish in the fish group, 4654 mg from krill oil in the krill group and 0 mg from HOSO in the control group (Table 1). The peroxide value (PV) was obtained to ensure that the krill oil was not oxidised, as oxidised *n*-3 supplements may negatively affect serum lipids^(^^41^^)^. The krill oil had a PV of 0·5 mEq/kg (Eurofins), indicating a low level of oxidation. It was not possible to assess the level of secondary oxidation products with the anisidine value because of the intense red colour of the oil. The control oil had added astaxanthin to achieve the same concentration as the krill oil (982 parts per million). The average fatty acid content in the three fish meals throughout the year and the fatty acid content of the encapsulated oils are given in the Supplementary information (Supplementary Tables S1 and S2).

**Table 1.** Average weekly intake (mg) of marine *n*-3 fatty acids from the intervention products (Mean values and ranges)

	Fish group		Control group	Krill group
	Mean	Range		
20 : 5*n*-3 (EPA)	1367	1184–1679	0	3118
22 : 5*n*-3 (DPA)	333	261–400	0	69
22 : 6*n*-3 (DHA)	2403	2249–2712	0	1466
Total *n*-3 long-chain PUFA	4103	3694–4791	0	4654

DPA, docosapentaenoic acid.

### Randomisation and blinding

The participants were randomised and stratified by sex and age above and below 50 years in a 1:1:1 ratio to one of the three intervention groups. Randomisation was performed by an external statistician, who was not involved in the recruitment or enrolment of participants, using block randomisation. The randomisation code was not revealed to the investigators who recruited and enrolled participants until the preliminary statistical analyses were completed.

The control group and the krill group were blinded for the participants and the investigators, except for one person working in the project that received the randomisation code and labelled the capsule containers with ID numbers. The capsules came in identical containers and were of equal size. Because of the added astaxanthin in the control oil, the oils had the same colour. At the end of the study visit, we asked the participants what group they thought they were randomised to. Five of twelve participants in the control group thought they received control oil and five of twelve participants in the krill group thought they received krill oil.

### Compliance and adverse events

Compliance was assessed by a compliance check list in the fish group and by capsule count in the krill and control groups. The number of capsules taken is expressed as a percentage of capsules defined for the 8-week intervention, and participants with compliance less than 80 % were considered as non-compliant. The estimated mean compliance was 100 % in the fish group, 97 % in the control group and 97 % in the krill group and the lowest level of estimated compliance was 83 % in the krill group. The plasma response to dietary fatty acids varies depending on the food matrix^(^^42^^)^, and in the present study, *n*-3 fatty acids were supplied as both fish and krill oil. Thus, we chose to use capsule count and compliance check list as the measure of compliance, and not plasma levels of EPA and DHA which have been reported as biomarkers and measure of compliance of intake of fatty fish and fish oil^(^^43^^,^^44^^)^.

Two subjects in the fish group reported adverse effects of the intervention (dizziness, vomiting, headache and fatigue), two in the control group (diarrhoea, constipation, bloating and nausea) and one in the krill group (belching, yellow urine and joint pain).

### Blood sampling

Blood samples were drawn at baseline and at the end of study visits after an overnight fast (≥12 h). Participants were instructed to avoid vigorous physical activity and alcohol consumption and to eat a low-fat dinner meal the day before blood sampling. Serum was obtained from silica gel tubes (Becton Dickinson Vacutainer Systems) that were kept at room temperature for at least 30 min until centrifugation (1500 ***g***, 15 min). Plasma was obtained from EDTA tubes (Becton Dickinson Vacutainer Systems), immediately placed on ice and centrifuged within 30 min (1000 ***g***, 4°C, 15 min) and stored at −80°C.

### Routine laboratory analysis

Fasting serum TAG, as well as total cholesterol, LDL-cholesterol and HDL-cholesterol, estimated glomerular filtration rate, alanine aminotransferase, γ-glutamyl transferase, glycated Hb, glucose, apoA1 and B, lipoprotein(a), high-sensitivity C-reactive protein, thyroid-stimulating hormone, free triiodothyronine (T3), free thyroxine (T4) and vitamin D were analysed by a standard clinical routine laboratory (Fürst Medical Laboratory, Oslo, Norway).

### Lipoprotein subclasses

Lipoprotein subclasses and lipid concentrations were quantified from EDTA plasma using a commercial high-throughput proton NMR metabolomics platform (Nightingale Health Ltd). Details of the experimentation and applications of the NMR metabolomics platform have been described previously^(^^36^^)^. The fourteen lipoprotein subclasses were defined by their average diameter: extremely large VLDL with a possible contribution of chylomicrons (>75 nm), five VLDL subclasses (64·0, 53·6, 44·5, 36·8 and 31·3 nm), intermediate-density lipoprotein (28·6 nm), three LDL subclasses (25·5, 23·0 and 18·7 nm) and four HDL subclasses (14·3, 12·1, 10·9 and 8·7 nm). The following components of the lipoprotein subclasses were quantified: phospholipids, cholesterol, cholesteryl esters, non-esterified cholesterol and TAG. The mean size for VLDL, LDL and HDL particles was calculated by weighting the corresponding subclass diameters with their particle concentrations.

### Plasma fatty acid analysis

To a sample of 40 µl EDTA plasma, 100 µl internal standard (triheptadecanoin) and 800 µl 3 m-methanolic HCl were added, mixed and heated at 50°C overnight. A quantity of 300 µl 3 m-KOH in water was added to the sample and the resulting fatty acid methyl esters were extracted to 500 µl hexane. GC analyses were performed using a 7890 N GC with a split/splitless injector, a 7683B automatic liquid sampler and a flame ionisation detector (Agilent Technologies). Separations were performed with a SP-2380 (30 m × 0·20 mm internal diameter × 0·25-μm film thickness) column from Supelco (Vitas Analytical Service).

### Statistics

Sample size calculation was based on previous studies^(^^32^^,^^45^^)^ where individuals with TAG between 1·7 and 4·0 mmol/l had a mean TAG level of 2·21 (sd 0·59) mmol/l. Based on a pilot study, we expected a 20 % decrease in fasting TAG following an intake of 800 mg/d marine *n*-3 fatty acids from krill oil in individuals with slightly elevated TAG^(^^46^^)^. The level of significance was set to 5 % and the power to 80 %. With an estimated drop-out rate of 10 %, a total of ninety-six participants were required in this study. However, due to difficulties in recruiting subjects with slightly elevated TAG, we were only able to randomise forty participants.

The Kruskal–Wallis test was used to test for overall differences between the three intervention groups for variables with a non-normal distribution. One-way ANOVA was used to test overall difference between groups for normally distributed variables. *P* < 0·05 was considered statistically significant. In the case of significant results, the Mann–Whitney–Wilcoxon test with Bonferroni corrected *P* values was used for *post hoc* pairwise comparisons. The Benjamini–Hochberg method was performed to control for multiple comparisons for secondary outcomes. Lipoprotein subclass variables were log_2_-transformed to enable visualisation of the data and the use of parametric tests. As the lipoprotein subclass measure was an explorative analysis, subclass variables were not adjusted for multiple comparisons. Missing data in this study were missing by random; hence, they were removed and analyses were performed in the remaining sample. All statistical analyses were per protocol and were performed in R^(^^47^^)^.

## Results

### Baseline characteristics

**Fig. 1.**
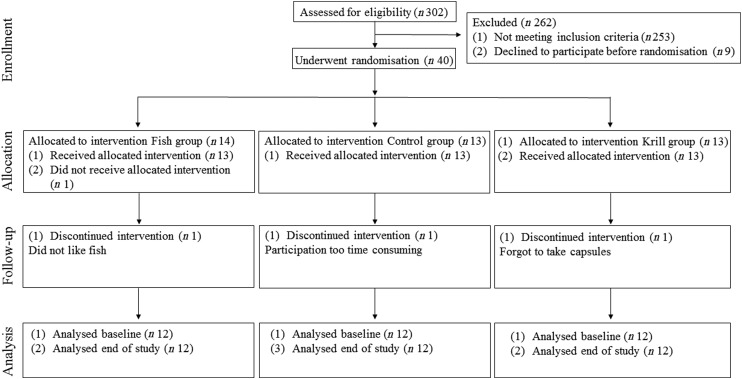
Flowchart of the study.

In total, 17 863 invitations were sent out by post, 302 subjects were screened for eligibility and forty subjects were randomised to receive the intervention. Participants lost during follow-up and the number of participants allocated to each of the intervention groups are shown in the flowchart (Fig. 1). A total of thirty-six participants completed the intervention period. There were twelve participants and 50 % men and women in each of the three intervention groups. The participants were 54 (interquartile range (IQR) 13) years in the fish group, 58 (IQR 5) years in the control group and 57 (IQR 16) years in the krill group and had baseline TAG of 1·52 (IQR 0·42), 1·81 (IQR 0·76) and 1·69 (IQR 0·51) mmol/l in the fish group, control group and krill group, respectively. The dietary intake at baseline did not differ between the intervention groups (Table 2). Clinical and biochemical baseline characteristics of the participants are shown in Table 3. There were four current smokers: two in the fish group and two in the krill group. One participant in each of the capsule groups used statins and antihypertensive medications and two participants in the control group used anti-inflammatory medications.

**Table 2.** Dietary intake at baseline (Mean values and standard deviations)

	Fish group (*n* 12)		Control group (*n* 12)		Krill group (*n* 12)		
	Mean	sd	Mean	sd	Mean	sd	P*
Energy (kJ)	11 186	3934	10 220	2869	10 533	3419	0·641
Protein (E%)	16·9	2·9	17·4	2·7	16·3	2·8	0·623
Fat (E%)	36·6	5·1	34·5	7·6	32·9	7·2	0·178
SFA (E%)	14·1	2·4	12·6	3·4	11·9	3·6	0·095
MUFA (E%)	13·5	2·3	12·5	3·0	12·1	2·8	0·214
PUFA (E%)	5·8	1·5	6·0	2·0	6·0	1·5	0·758
Carbohydrates (E%)	41·7	6·2	41·9	9·4	45·2	9·1	0·307
Fibre (E%)	2·2	0·4	2·7	0·7	2·6	1·0	0·189
Sugar (E%)	7·7	4·4	5·8	3·2	5·8	3·0	0·193
Alcohol (E%)	2·7	1·8	3·5	3·1	3·0	3·1	0·747

E%, percentage of total energy.

* Differences between groups were tested with one-way ANOVA.

**Table 3.** Biochemical and clinical parameters at baseline and at the end of the study (*n* 36) (Median values and interquartile ranges (IQR))

	Fish group (*n* 12)‡				Control group (*n* 12)				Krill group (*n* 12)§				
	Baseline		End of study		Baseline		End of study		Baseline		End of study		
	Median	IQR	Median	IQR	Median	IQR	Median	IQR	Median	IQR	Median	IQR	*P*ǁ
Total cholesterol (mmol/l)	5·8	0·9	5·9	1·2	5·7	1·2	6·0	1·3	6·2	1·3	6·3	1·3	0·590
HDL-cholesterol (mmol/l)	1·4	0·3	1·3	0·3	1·3	0·4	1·3	0·3	1·4	0·2	1·4	0·3	0·674
LDL-cholesterol (mmol/l)	3·9	0·9	3·7	0·9	3·8	2·0	4·2	1·5	4·0	1·0	3·9	0·9	0·700
ApoA1 (g/l)	1·8	0·2	1·7	0·1	1·8	0·5	1·6	0·2	1·6	0·3	1·7	0·2	0·255
ApoB (g/l)	1·2	0·2	1·1	0·3	1·2	0·4	1·2	0·4	1·3	0·3	1·3	0·3	0·212
ApoB/ApoA1	0·7	0·2	0·7	0·3	0·7	0·3	0·7	0·2	0·8	0·1	0·8	0·1	0·866
Lp(a) (mg/l)	106	129	113	109	133	139	124	133	100	101	100	64	0·974
BMI (kg/m^2^)	28·5	5·5	28·7	5·3	28·0	5·0	27·7	5·1	26·4	5·1	26·1	5·4	0·046†
Systolic BP (mmHg)	127	17	123	26	122	28	132	21	122	11	126	20	0·362
Diastolic BP (mmHg)	82	12	75	19	78	12	78	13	76	9	80	8	0·356
hsCRP (mg/l)	1·3	0·8	1·4	1·0	1·3	2·6	1·3	2·4	1·3	2·0	1·9	2·6	0·168
Vitamin D (nmol/l)	56	26	72	31	72	33	78	14	76	30	67	16	0·024*
HbA1c (%)	5·7	0·5	5·6	0·5	5·4	0·3	5·4	0·0	5·4	0·2	5·4	0·2	0·673
Glucose (mmol/l)	5·3	0·5	5·4	0·5	5·2	0·3	5·5	0·4	5·6	0·6	5·3	0·5	0·024†
TSH (mU/l)	2·0	1·3	1·5	1·1	1·7	0·7	2·0	1·2	1·3	1·0	1·5	1·0	0·193
Free T4 (pmol/l)	15·1	4·0	16·0	2·6	15·1	3·5	15·8	3·5	16·3	2·7	16·9	1·4	0·589
Free T3 (pmol/l)	5·3	0·7	5·1	0·4	4·9	0·4	5·1	0·4	5·2	0·8	5·1	0·5	0·144
Creatinine (μmol/l)	67	17	69	13	81	19	78	20	74	15	72	17	0·761
eGFR (ml/min per 1·73 m^2^)	93	16	94	20	82	21	87	16	91	19	89	20	0·891
ALAT (U/l)	29	17	28	7	25	7	20	13	22	10	22	15	0·303
γ-GT (U/l)	31	27	30	27	24	7	21	9	24	13	25	49	0·014†

Lp(a), lipoprotein a; BP, blood pressure; hsCRP, high-sensitivity C-reactive protein; HbA1c, glycosylated Hb; TSH, thyroid-stimulating hormone; T4, thyroxine; T3, triiodothyronine; eGFR, estimated glomerular filtration rate; ALAT, alanine aminotransferase; γ-GT, γ-glutamyl transferase.

* Significantly different between the fish group and krill group (*P* < 0·05).

† Significantly different between the krill group and control group (*P* < 0·05).

‡ *n* 11 for apoA1, apoB, Lp(a), HbA1c, glucose, vitamin D, creatinine, ALAT and γ-GT. *n* 9 for eGFR.

§ *n* 11 for HbA1c and ALAT.

ǁ Overall difference between the three intervention groups was tested with the Kruskal–Wallis test; *P* values are given in the Table. Significant results were further tested with the Mann–Whitney–Wilcoxon test for pairwise comparisons, and significant results are indicated with symbols.

### Effects on fasting serum TAG

**Fig. 2.**
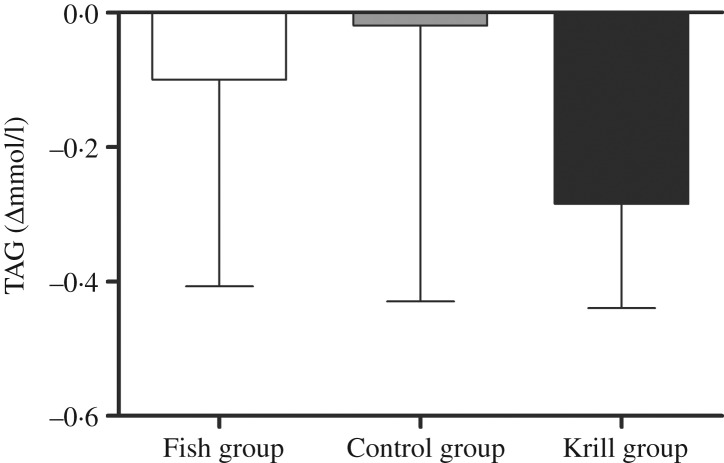
Change in fasting serum TAG from baseline to the end of the study. Values are medians, with interquartile ranges represented by vertical bars. Kruskal–Wallis test for overall difference between groups: *P* = 0·59.

**Fig. 3.**
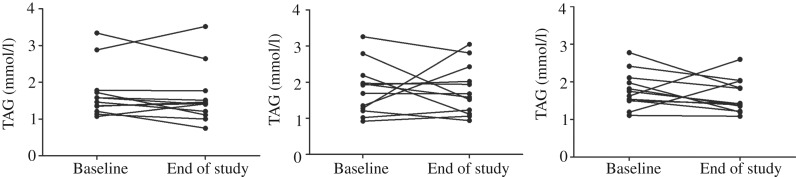
Individual changes in fasting serum TAG from baseline to the end of the study in the fish (a), control (b) and krill (c) groups.

The primary outcome in this randomised controlled trial was fasting serum TAG. There was a reduction in fasting TAG of 0·29 mmol/l (17 %) in the krill group, 0·1 mmol/l (7 %) in the fish group and 0·02 mmol/l (1 %) in the control group; however, there were no significant differences within or between the groups (Fig. 2). Nonetheless, in the krill group, there was a reduction in TAG from baseline to the end of the study in all except two participants (Fig. 3(c)), while there was a less clear pattern in the change in fasting TAG in the fish group and the control group (Fig. 3(a) and (b)).

### Lipoprotein subclasses

**Fig. 4.**
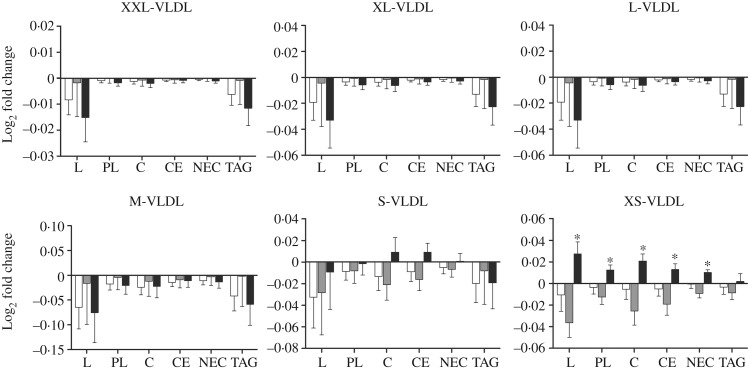
Log_2_-transformed fold change in VLDL subclasses for total lipids (L), phospholipids (PL), cholesterol (C), cholesteryl esters (CE), non-esterified cholesterol (NEC) and TAG: (a) extremely large VLDL (XXL-VLDL), (b) very large VLDL (XL-VLDL), (c) large VLDL (L-VLDL), (d) medium VLDL (M-VLDL), (e) small VLDL (S-VLDL) and (f) very small VLDL (XS-VLDL). (□), Fish group (*n* 11); (■), krill group (*n* 12); (▒), control group (*n* 12). Values are means, with standard errors represented by vertical bars. Overall significant differences in the changes were determined by one-way ANOVA. * *P* < 0·05.

**Fig. 5.**
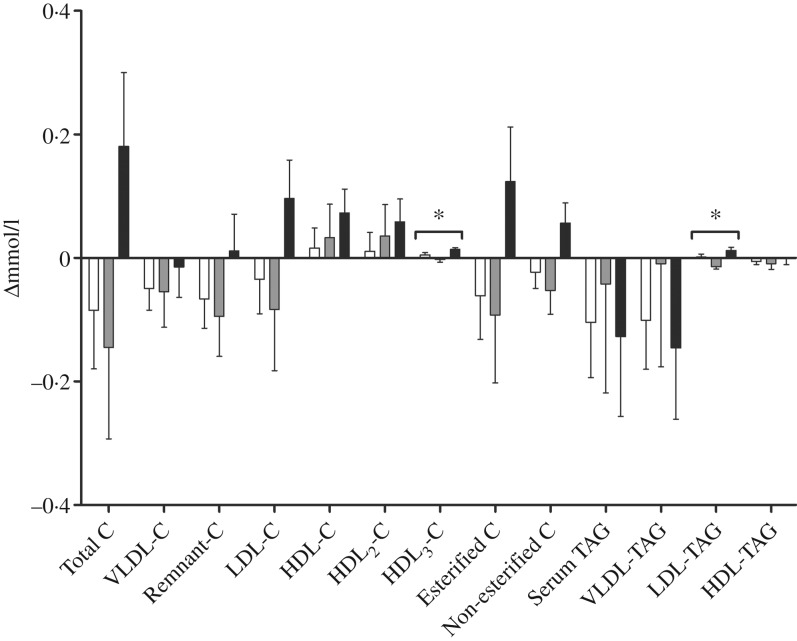
Change in fractions of TAG and cholesterol (C) measured with NMR spectroscopy in groups receiving either fish (□; *n* 11), krill oil (■; *n* 12) or control oil (▒; *n* 12). Values are means, with standard errors represented by vertical bars. Overall significant differences in the changes were determined by one-way ANOVA. * *P* < 0·05.

We performed a comprehensive analysis of lipoprotein subclasses and their lipid constituents using an NMR platform as an explorative part of this study to further understand the role of *n*-3 fatty acids from different sources. The concentration of total lipids (*P* = 0·007), phospholipids (*P* = 0·015), cholesterol (*P* = 0·009), cholesteryl esters (*P* = 0·022) and non-esterified cholesterol (*P* = 0·002), but not TAG, in the smallest VLDL subclass (XS-VLDL) significantly increased in the krill group compared with the fish group and the control group (Fig. 4). Furthermore, the particle concentration of XS-VLDL increased significantly in the krill group compared with the fish group and the control group (*P* = 0·007, not shown). There was an increase in HDL_3_-cholesterol and LDL-TAG in the krill group and the fish group, (Fig. 5) and the overall difference between the three groups was significant (*P* = 0·009 and *P* = 0·002, respectively). Finally, we observed that the reduction in serum TAG to a large extent corresponds to the reduction in VLDL-TAG (Fig. 5).

### Biochemical and clinical parameters

After the 8-week intervention, there was no difference between the groups in the change in total cholesterol, LDL-cholesterol or HDL-cholesterol (Table 3). However, the level of fasting glucose decreased from 5·6 to 5·3 mmol/l in the krill group, while there was an increase in the two other groups. The difference between the three groups was significant and pairwise comparisons revealed that the krill group was significantly different from the control group (*P* = 0·01). The level of vitamin D increased from 56 to 72 nmol/l in the fish group, while there was a small increase from 72 to 78 nmol/l in the control group and a decrease from 76 to 67 nmol/l in the krill group. The overall difference between the three groups was significant, and pairwise comparisons showed that the increase in the fish group was significantly different from the decrease in the krill group (*P* = 0·004). The difference in change in vitamin D between the groups remained significant after adjusting for seasonal effects on vitamin D levels (not shown). The level of γ-glutamyl transferase, a marker of liver disease, increased in the krill group while it decreased in the fish group and the control group. The overall difference was significant, and pairwise comparisons showed that the increase in the krill group was significantly different from the control group (*P* = 0·007). There was a difference in change in BMI between the three groups, although this was a result of one outlier in the krill group who increased BMI from baseline to the end of the study. The increase in BMI in the krill group was significantly different from that of the control group (*P* = 0·049). Removing the BMI outlier did not alter any biochemical, clinical, plasma fatty acid or lipoprotein subclass results, except BMI, which was no longer significantly different between the groups. None of the changes in biochemical and clinical parameters was significant after correcting for multiple comparisons (not shown). There were no changes in the physical tests from baseline to the end of the study (not shown).

### Plasma fatty acids

The plasma levels of fatty acids were measured at baseline and after the intervention. The level of EPA and DHA increased in the fish group and krill group, whereas docosapentaenoic acid (DPA) increased only in the krill group (Table 4). The overall differences between the three intervention groups were significant for EPA (*P* < 0·0001), DPA (*P* < 0·001) and DHA (*P* < 0·001). *Post hoc* analyses showed that the change in EPA was significantly different in all pairwise comparisons. The increase in DPA in the krill group was significantly different from the fish group and the control group and the increases in DHA in the fish group and the krill group were significantly different from the control group. The increase in plasma marine *n*-3 fatty acids in the krill group was accompanied by a reduction in oleic acid. The overall difference was significant between the groups (*P* = 0·011) (Table 4). The calculated weekly intake of EPA from the intervention was higher in the krill group than in the fish group (Table 1), and *post hoc* analysis revealed that this was reflected by a significantly greater increase in plasma EPA in the krill group compared with the fish group (*P* = 0·002) (Table 4). However, the calculated weekly intake of DHA and DPA from the intervention was higher in the fish group than in the krill group, but the increase in plasma DHA was not different between the krill group and fish group (*P* = 0·25). Furthermore, plasma DPA increased in the krill group while it decreased slightly in the fish group, and the difference between the two groups was significant (*P* = 0·001) (Table 4). All significant plasma fatty acid results remained significant after adjusting for multiple comparisons (not shown).

**Table 4.** Plasma fatty acids (% weight) at baseline and at the end of the study (Median values and interquartile ranges (IQR))

	Fish group (*n* 12)				Control group (*n* 12)				Krill group (*n* 12)				
	Baseline		End of study		Baseline		End of study		Baseline		End of study		
Plasma fatty acids (% wt)	Median	IQR	Median	IQR	Median	IQR	Median	IQR	Median	IQR	Median	IQR	*P*§
14 : 0 (myristic acid)	1·10	0·33	1·10	0·53	0·90	0·33	0·95	0·53	0·90	0·33	0·90	0·43	0·765
16 : 0 (palmitic acid)	20·75	1·78	21·10	2·43	20·55	2·05	21·10	1·73	20·65	1·80	20·60	2·00	0·993
18 : 0 (stearic acid)	6·65	0·75	6·75	0·25	6·50	0·88	6·55	1·00	6·45	0·43	6·80	0·60	0·457
18 : 1*n*-9 (oleic acid)	22·05	2·53	21·20	2·20	22·80	3·73	23·30	4·53	23·90	1·97	21·45	2·10	0·0109*
18 : 2*n*-6 (linoleic acid)	26·10	3·13	26·10	4·58	25·10	5·58	24·25	4·50	26·00	3·15	26·85	4·30	0·998
18 : 3*n*-6	0·40	0·20	0·30	0·13	0·40	0·13	0·40	0·20	0·40	0·13	0·30	0·10	0·260
20 : 2*n*-6	0·20	0·00	0·20	0·00	0·20	0·00	0·20	0·00	0·20	0·00	0·20	0·00	0·493
20 : 3*n*-6	1·50	0·15	1·35	0·38	1·40	0·25	1·35	0·23	1·40	0·30	1·30	0·20	0·837
20 : 4*n*-4 (AA)	5·65	1·53	5·50	1·78	5·65	0·93	5·70	1·95	5·45	0·73	5·10	0·48	0·315
Total *n*-6 fatty acids	34·25	5·20	33·95	6·63	32·50	5·65	32·10	6·13	33·40	2·35	34·05	5·60	0·796
18 : 3*n*-3 (ALA)	0·75	0·23	0·65	0·20	0·80	0·20	0·75	0·23	0·70	0·30	0·80	0·30	0·485
20 : 5*n*-3 (EPA)	1·10	0·35	1·25	0·85	1·10	0·35	1·05	0·53	0·95	0·35	2·50	0·68	0·00004*†‡
22 : 5*n*-3 (DPA)	0·60	0·13	0·55	0·10	0·50	0·10	0·55	0·10	0·60	0·10	0·70	0·13	0·0003*‡
22 : 6*n*-3 (DHA)	2·10	0·83	2·70	0·78	2·25	0·53	2·05	0·88	2·10	0·83	2·75	0·58	0·0008†‡
Total *n*-3 fatty acids	4·45	1·25	5·30	2·00	4·70	0·90	4·45	1·38	4·40	1·08	6·25	1·40	0·00007*†‡

AA, arachidonic acid; ALA, α-linolenic acid; DPA, docosapentaenoic acid.

* Significantly different between the fish group and krill group (*P* < 0·05).

† Significantly different between the fish group and control group (*P* < 0·05).

‡ Significantly different between the krill group and control group (*P* < 0·05).

§ Overall difference between the three intervention groups was tested with the Kruskal–Wallis test; *P* values are shown in table. Significant results were further tested with the Mann–Whitney–Wilcoxon test for pairwise comparisons, and significant results are indicated with symbols.

## Discussion

In this study, we have found that intake of krill oil, or lean and fatty fish do not significantly reduce serum TAG compared with a control in individuals with slightly elevated TAG. Total lipids, phospholipids, cholesterol, cholesteryl esters and non-esterified cholesterol in the smallest VLDL subclass increased significantly in the krill group compared with in the fish and control groups. In addition, HDL_3_-cholesterol and LDL-TAG increased in the krill group and the fish group and the overall differences between the three intervention groups were significant. Furthermore, we found a reduction in fasting glucose in the krill group, an increase in vitamin D in the fish group and an increase in plasma marine *n*-3 fatty acids in the fish group and the krill group.

Krill oil did not reduce fasting TAG in the present study; however, all except two participants in the krill group had a reduction in fasting TAG with a less clear pattern in TAG change in the fish and control groups. Other krill oil studies have reported a TAG-lowering effect in human subjects. In a study by Bunea *et al*.^(^^29^^)^, krill oil with an unknown concentration of *n*-3 fatty acids, in high krill oil doses (2–3 g/d), significantly reduced TAG within the group, while lower krill oil doses (1–1·5 g/d) reduced fasting TAG non-significantly^(^^29^^)^. Krill oil was also shown to reduce fasting TAG by 10 % in a pooled analysis of four different krill oil doses in subjects with slightly elevated TAG^(^^48^^)^. In a pilot study without a control group, TAG was reduced by 12 % after supplementation with 833  mg EPA + DHA per d from krill oil^(^^46^^)^. In addition, krill oil containing krill powder reduced TAG by 20 % in a 24-week study in hypertriacylglycerolaemic obese men^(^^49^^)^. However, other krill oil studies did not see a TAG-reducing effect. These studies have included subjects with low baseline TAG and were of short duration^(^^30^^–^^32^^)^, although there was a TAG-reducing effect in participants with the highest baseline TAG in the study by Ulven *et al*.^(^^32^^)^. Hence, well-designed studies with more participants with elevated serum TAG and perhaps longer duration are needed to establish the effect of krill oil on TAG.

In this study, we found large individual differences in the TAG response to krill oil and fish. These results are in line with, for example, the Fatty Acid Sensor (FAS) study and the Fish Oil Intervention and Genotype (FINGEN) study, where about 30 % of the participants did not have reduced plasma TAG after *n*-3 supplementation^(^^50^^,^^51^^)^. These inter-individual differences are partly the result of SNP, although differences in genetic loci cannot explain all the observed variance^(^^52^^)^. Hence, differing genotypes could explain some of the heterogeneity in the TAG response to krill oil supplementation and intake of fish in this study, and the interventions might thus be more beneficial for subjects with a certain genetic profile.

Although there were no significant changes in fasting TAG, there was a tendency towards a decrease in total lipids and TAG in the largest VLDL subclasses following fish and krill oil consumption. The significant increase in all lipid constituents in XS-VLDL, except TAG, in the krill group might thus suggest an accumulation of the smallest VLDL subclass because of a depletion of TAG in the larger VLDL subclasses. This may have been a result of increased lipoprotein lipase activity, which has previously been described after *n*-3 supplementation^(^^53^^)^. Non-esterified cholesterol and phospholipids are shed from TAG-rich lipoproteins after TAG depletion by lipoprotein lipase and associate with discoidal HDL particles to form HDL_3_ particles^(^^54^^)^. Thus, an increased lipoprotein lipase activity might also explain the significant increase in HDL_3_-cholesterol in the krill group.

We also observed a significant reduction in fasting blood glucose in the krill group, compared with the control group, from 5·6 to 5·3 mmol/l. Glucose level is strongly associated with CVD risk^(^^55^^,^^56^^)^. The finding in this study is in agreement with previous work showing that krill oil reduces blood glucose in some animal models^(^^57^^–^^59^^)^ and in human subjects^(^^29^^)^. Studies of hepatic gene expression in mice have demonstrated that krill oil and krill powder supplementation down-regulates genes involved in gluconeogenesis^(^^60^^,^^61^^)^. This can be a possible explanation for the observed effects on fasting glucose, although this needs to be confirmed by gene expression analysis in human subjects. The effect of fish or fish oil consumption on glucose levels, however, is less clear^(^^62^^)^.

The level of vitamin D increased significantly in the fish group compared with the krill group, from 56 to 72 nmol/l. Low vitamin D levels have been associated with CVD risk in several observational studies^(^^63^^–^^66^^)^. An optimal vitamin D level is regarded to be above 75 nmol/l, while levels below 50 nmol/l are regarded as vitamin D deficiency^(^^67^^)^. Hence, it can be argued that the change we observed in this study is clinically relevant. A meta-analysis of randomised controlled trials investigating fish intake and vitamin D status found an average increase in vitamin D of 4·4 nmol/l^(^^68^^)^. The increase we observed in this study of 16 nmol/l might on one hand be a result of a relatively low baseline level in the fish group. On the other hand, salmon and mackerel are thought to be good sources of vitamin D, although the content may vary along with seasonal variations in fat content^(^^69^^)^.

Finally, the level of plasma marine *n*-3 fatty acids significantly increased in the krill group and the fish group, but not in the control group. This is as expected, because plasma *n*-3 fatty acids have been suggested as a biomarker of intake of fish and *n*-3 supplements^(^^70^^)^. The intake of EPA from the intervention in the krill group was higher than in the fish group, resulting in a more pronounced increase in plasma EPA. The intake of DHA from the intervention in the fish group was higher than in the krill group, 2403 and 1466 mg/week, respectively. Interestingly, the increase in plasma DHA in the krill group was similar to the increase in the fish group. Similarly, the intake of DPA from the intervention was higher in the fish group than in the krill group, but there was an increase in plasma DPA in the krill group only. This might imply a greater bioavailability of marine *n*-3 fatty acids from krill oil than from fish, although this was not directly measured, supporting previous studies suggesting a high bioavailability of krill oil^(^^33^^)^.

The major limitation of this study is that it was not powered to detect changes in the primary outcome, fasting TAG, because of difficulties in recruiting participants. One other limitation is that this was not a fully controlled dietary intervention. Even though we asked the participants to maintain their habitual diet and level of physical activity, we cannot know if these factors affected the outcomes of this study. Furthermore, the HOSO we used as a control is not a true placebo because it is not a biologically inactive compound. Nonetheless, there were no changes in plasma fatty acids in the control group, suggesting that the fatty acid contribution from the HOSO is minor compared with the fatty acid intake from the diet. Despite these limitations, this study is strengthened by the high compliance. Overall compliance was 98 %, and no participant had compliance below 80 %. Furthermore, we achieved successful blinding in the krill and control groups.

In conclusion, we observed that consumption of lean and fatty fish, according to dietary recommendations, and supplementation with krill oil with a similar content of *n*-3 fatty acids have beneficial health effects. Although krill oil had a more beneficial effect on blood glucose than fish, fish is also a good source of other health-beneficial nutrients, including vitamin D.

## Supplementary material

For supplementary material accompanying this paper visit https://doi.org/10.1017/jns.2017.64 click here to view supplementary material
