# Supplementary material for: Effects of krill oil and lean and fatty fish on cardiovascular risk markers: a randomised controlled trial
Source: J Nutr Sci. 2018 Jan 17;7:e3. doi: 10.1017/jns.2017.64 (PMC5773922; doi:10.1017/jns.2017.64)
Supplement: Supplementary file 1 [file S2048679017000647sup001.doc]

**Supplementary tables**

Supplementary Table S1. Fatty acid composition of the fish meals

| Fatty acid (mg/portion) | Salmon (140 g) | Cod in leek sauce (550 g, 26% cod) | Mackerel in tomato sauce (110 g, 60% mackerel) |
| --- | --- | --- | --- |
| 14:0 | 264.6 | 331.0 | 874.2 |
| 15:0 | 24.4 | 31.6 | 66.8 |
| 16:0 | 1229.4 | 1088.9 | 1967.2 |
| 18:0 | 324.5 | 382.4 | 387.9 |
| 20:0 | 41.4 | 5.8 | 45.1 |
| 22:0 | 18.4 | 3.0 | 22.6 |
| 24:0 | 11.7 | 3.3 | 0.0 |
| Total saturated | 1905.1 | 1885.4 | 3363.9 |
| 16:1n-9 | 28.0 | 7.6 | 35.2 |
| 16: n-7 | 304.4 | 57.4 | 591.9 |
| 18:1n-9 | 4822.6 | 720.7 | 3898.9 |
| 18:1n-7 | 398.4 | 89.3 | 433.6 |
| 20:1n-11 | 26.8 | 13.2 | 111.1 |
| 20:1n-9 | 432.1 | 20.6 | 1213.2 |
| 20:1n-7 | 21.1 | 0.0 | 31.3 |
| 22:1n-11 | 223.5 | 6.9 | 1869.6 |
| 22:1n-9 | 64.0 | 2.9 | 133.6 |
| 24:1n-9 | 50.3 | 4.3 | 127.5 |
| Total monounsaturated | 6371.1 | 921.3 | 8445.9 |
| 18:2n-6 | 1599.9 | 78.3 | 1089.9 |
| 18:3n-6 | 11.4 | 2.7 | 27.6 |
| 20:2n-6 | 130.0 | 2.3 | 40.6 |
| 20:3n-6 | 24.3 | 4.1 | 16.6 |
| 20:4n-6 | 39.2 | 13.4 | 57.8 |
| 22:4n-6 | 9.7 | 0.0 | 0.0 |
| 22:5n-6 | 11.5 | 2.6 | 40.5 |
| Total n-6 PUFA | 1816.0 | 92.6 | 1269.7 |
| 18:3n-3 | 668.1 | 23.3 | 580.5 |
| 18:4n-3 | 74.0 | 6.7 | 572.0 |
| 20:3n-3 | 60.7 | 0.0 | 28.0 |
| 20:4n-3 | 94.2 | 4.0 | 130.3 |
| 20:5n-3 (EPA) | 361.1 | 88.0 | 917.6 |
| 21:5n-3 | 20.7 | 2.7 | 61.4 |
| 22:5n-3 (DPA) | 161.7 | 9.6 | 162.1 |
| 22:6n-3 (DHA) | 610.6 | 228.8 | 1563.7 |
| Total n-3 PUFA | 2045.5 | 360.7 | 4015.6 |
| 16:2 | 25.6 | 3.2 | 40.1 |
| 16:3 | 19.9 | 3.3 | 21.7 |
| 16:4 | 17.4 | 0.0 | 49.2 |
| Total PUFA | 3927.6 | 455.8 | 5396.3 |
| Total | 12203.9 | 3262.5 | 17206.0 |

EPA, eicosapentaenoic acid, DPA, docosapentaenoic acid, DHA, docosahexaenoic acid

Supplementary Table S2. Fatty acid composition of the krill oil and high-oleic sunflower oil

| Fatty acid (g/100g oil) | High-oleic sunflower oil | Krill oil |
| --- | --- | --- |
| 14:0 | 0.0 | 4.9 |
| 15:0 | 0.0 | 0.2 |
| 16:0 | 3.2 | 12.6 |
| 18:0 | 2.5 | 0.8 |
| 20:0 | 0.2 | 0.0 |
| 22:0 | 0.8 | 0.1 |
| 24:0 | 0.3 | 0.0 |
| Total saturated | 6.9 | 18.7 |
| 16:1n-9 | 0.0 | 0.1 |
| 16:1n-7 | 0.1 | 4.2 |
| 18:1n-9 | 73.4 | 6.6 |
| 18:1n-7 | 0.6 | 4.1 |
| 20:1n-11 | 0.0 | 0.1 |
| 20:1n-9 | 0.3 | 0.5 |
| 20:1n-7 | 0.0 | 0.2 |
| 22:1n-11 | 0.0 | 0.1 |
| 22:1n-9 | 0.1 | 0.4 |
| 24:1n-9 | 0.0 | 0.1 |
| Total monounsaturated | 74.5 | 16.3 |
| 18:2n-6 | 8.0 | 0.9 |
| 18:3n-6 | 0.0 | 0.1 |
| 20:2n-6 | 0.0 | 0.0 |
| 20:3n-6 | 0.0 | 0.4 |
| 20:4n-6 | 0.0 | 0.2 |
| 22:4n-6 | 0.0 | 0.0 |
| 22:5n-6 | 0.0 | 0.0 |
| Total n-6 PUFA | 8.0 | 1.8 |
| 18:3n-3 | 0.2 | 0.4 |
| 18:4n-3 | 0.0 | 1.4 |
| 20:3n-3 | 0.0 | 0.1 |
| 20:4n-3 | 0.0 | 0.2 |
| 20:5n-3 (EPA) | 0.0 | 11.1 |
| 21:5n-3 | 0.0 | 0.3 |
| 22:5n-3 (DPA) | 0.0 | 0.2 |
| 22:6n-3 (DHA) | 0.0 | 5.2 |
| Total n-3 PUFA | 0.2 | 19.0 |
| 16:2 | 0.0 | 0.4 |
| 16:3 | 0.0 | 0.1 |
| 16:4 | 0.0 | 0.5 |
| Total PUFA | 8.2 | 21.8 |
| Total | 89.7 | 56.7 |

EPA, eicosapentaenoic acid, DPA, docosapentaenoic acid, DHA, docosahexaenoic acid
